# Optimizing Perioperative Nutrition in Elective Gastrointestinal Surgery: An ERAS-Focused Narrative Review

**DOI:** 10.3390/nu18060984

**Published:** 2026-03-19

**Authors:** Maria Alexandra Brăgaru, Alin Kraft, Cosmin-Alec Moldovan, Adina-Diana Moldovan, Adam Răzvan, Daniel Cochior, Andrei Luca, Delia Nica-Badea, Ștefan Eugen Chirsanov Capanu, Elena Rusu

**Affiliations:** 1 Department of Preclinical Sciences, Faculty of Medicine, Titu Maiorescu University of Bucharest, 031593 Bucharest, Romania; maria.bragaru@yahoo.com (M.A.B.); diana.moldovan@prof.utm.ro (A.-D.M.); lc_andrei@yahoo.com (A.L.); elenarusu98@yahoo.com (E.R.); 2 Doctoral School in Medicine, Titu Maiorescu University of Bucharest, 031593 Bucharest, Romania; 3 Department of Medical-Surgical and Prophylactic Disciplines, Faculty of Medicine, Titu Maiorescu University of Bucharest, 031593 Bucharest, Romania; cosmin.moldovan@prof.utm.ro (C.-A.M.); daniel.cochior@prof.utm.ro (D.C.); 4 Department of General Surgery, “General Doctor Aviator Victor Anastasiu” National Aeronautical and Space Medicine Institute, 010242 Bucharest, Romania; 5 Department of General Surgery, Witting Clinical Hospital, 010243 Bucharest, Romania; 6 MedLife SA, 010719 Bucharest, Romania; 7 Department of Orthopedics and Traumatology, Elias Emergency University Hospital, 011461 Bucharest, Romania; adamrazvan31@gmail.com; 8 Department of First Aid and Disaster Medicine, Faculty of Medicine, Titu Maiorescu University of Bucharest, 031593 Bucharest, Romania; 9 Department of General Surgery, Monza Hospital, 021967 Bucharest, Romania; 10 Health and Mobility Department, Medicinal and Behavioral Sciences Faculty, Constantin Brâncuși University, 210135 Târgu-Jiu, Romania; nicabadeadelia@yahoo.com; 11 Department of Specialties in Dental Medicine, Faculty of Dental Medicine, Titu Maiorescu University of Bucharest, 031593 Bucharest, Romania; stefan.chirsanov@prof.utm.ro

**Keywords:** elective gastrointestinal surgery, perioperative nutrition, ERAS, malnutrition, sarcopenia, nutritional prehabilitation, carbohydrate loading, early postoperative feeding

## Abstract

**Background/Objectives:** Perioperative malnutrition, sarcopenia, and reduced functional reserve are frequent in adults undergoing elective gastrointestinal (GI) surgery and are associated with higher postoperative morbidity and delayed recovery. Enhanced Recovery After Surgery (ERAS) pathways incorporate nutrition-focused elements, but reported effects vary across procedures, protocols, and baseline risk. This review aims to summarize and critically appraise current evidence on perioperative nutritional strategies within ERAS-focused elective GI care, including risk identification, nutritional prehabilitation (oral nutritional supplements and immunonutrition), preoperative carbohydrate loading, early postoperative feeding, and selected microbiome-directed adjuncts. **Methods:** This narrative literature review was informed by a focused search of PubMed/MEDLINE and Scopus (2010–early 2026), supplemented by targeted screening of relevant clinical practice guidelines and consensus statements (e.g., ESPEN). Evidence was interpreted by hierarchy (guidelines/meta-analyses, randomized trials, observational studies) and discussed with attention to heterogeneity in surgical populations, intervention definitions (composition, timing, duration), and endpoint reporting. **Results:** Early nutritional risk screening is consistently supported to identify malnutrition and sarcopenia and to trigger tailored optimization plans. Perioperative oral nutritional supplementation, particularly when started preoperatively and continued postoperatively, is frequently associated with improved intake and reduced infectious morbidity in malnourished or at-risk patients, though effect sizes vary. Immunonutrition shows potential benefit in selected high-risk settings but remains formulation- and timing-dependent. Carbohydrate loading is generally endorsed within ERAS and may reduce insulin resistance and improve patient comfort, while impacts on major clinical outcomes are context-dependent. Early oral/enteral feeding is feasible in many elective GI procedures and may accelerate gastrointestinal recovery without increasing major complications when implemented with structured advancement and appropriate patient selection. Probiotics/synbiotics show the most consistent signals in colorectal surgery, with strain-specific effects and important safety boundaries in immunocompromised or critically ill patients. **Conclusions:** Perioperative nutritional optimization is a core component of elective GI surgical care within ERAS pathways. Benefits are most reproducible in higher-risk patients and when interventions are integrated into high-compliance multidisciplinary programs. Future research should prioritize procedure-specific, risk-stratified trials with standardized interventions and clinically meaningful endpoints.

## 1. Introduction

Elective gastrointestinal (GI) surgery encompasses planned, non-emergency procedures performed to diagnose or treat disorders of the digestive system, including conditions affecting the esophagus, stomach, liver, pancreas, and intestines. It is commonly undertaken for obesity, gastrointestinal malignancy, severe gastroesophageal reflux disease (GERD), and structural abnormalities, and includes operations such as gastrectomy, Roux-en-Y gastric bypass, colorectal resections, hernia repair, and bariatric procedures [1]. Across this spectrum, elective GI surgery elicits a pronounced surgical stress response characterized by hypermetabolism, insulin resistance, systemic inflammation, and accelerated skeletal muscle catabolism [2]. These metabolic and inflammatory perturbations contribute to clinically relevant postoperative complications, including surgical site infection, anastomotic leak, prolonged ileus, impaired wound healing, and delayed functional recovery [3]. In parallel, a substantial proportion of patients present preoperatively with malnutrition, cancer cachexia, or sarcopenia, all of which are independently associated with increased morbidity, longer length of stay, higher readmission rates, and greater healthcare expenditure, thereby positioning perioperative nutrition as a modifiable determinant of surgical resilience rather than a purely adjunctive therapy [4]. In contemporary perioperative practice, nutritional optimization is increasingly integrated into Enhanced Recovery After Surgery (ERAS) pathways and is supported by guidance from the European Society for Clinical Nutrition and Metabolism (ESPEN) [5]. Core components include systematic preoperative nutritional evaluation and risk stratification using validated screening tools, recognition of sarcopenia, and implementation of nutritional prehabilitation strategies such as high-protein oral nutritional supplementation, selected immunonutrition protocols, and targeted micronutrient replacement. Additional ERAS-aligned measures include preoperative carbohydrate loading as metabolic preparation aimed at attenuating insulin resistance and improving patient comfort while avoiding prolonged fasting, as well as early postoperative oral intake and/or enteral nutrition (EN) to limit underfeeding, preserve lean body mass, and support gastrointestinal function. Notably, the magnitude of benefit reported for individual interventions may vary across surgical procedures and baseline risk profiles, underscoring the need to interpret the literature within a pathway-based framework. Current evidence informing perioperative nutrition within ERAS pathways is derived from clinical practice guidelines and a heterogeneous body of comparative studies, with variability in intervention definitions (composition, timing, duration), surgical case-mix, and outcome reporting. As a result, several aspects remain debated, including the consistency of benefit from immunonutrition across procedures, the impact of carbohydrate loading on major clinical endpoints beyond metabolic outcomes, and the optimal timing and advancement of early postoperative feeding in higher-risk resections. Overall, the literature most consistently supports risk-stratified nutritional optimization within high-compliance ERAS programs, while emphasizing that effects are procedure- and protocol-dependent.

The aim of this narrative review is to analyze the evidence underpinning these perioperative nutritional strategies in elective upper and lower GI surgery, focusing on outcomes that matter to patients and clinicians, including infectious complications, anastomotic integrity, ileus, functional recovery, feeding tolerance, and patient-centered measures.

## 2. Materials and Methods

This article is a narrative literature review addressing perioperative nutritional strategies in adult patients undergoing elective gastrointestinal surgery, with emphasis on nutritional screening and diagnosis (malnutrition and sarcopenia), nutritional prehabilitation (including oral nutritional supplements and immunonutrition), preoperative carbohydrate loading, and early postoperative oral/enteral feeding within enhanced recovery pathways. A focused literature search was conducted in PubMed/MEDLINE and Scopus (last search: 15 February 2026), supplemented by targeted screening of relevant clinical practice guidelines and consensus statements from major societies (including ESPEN and ERAS Society guidance). Search strings combined controlled vocabulary and free-text terms, including (“perioperative nutrition” OR “nutritional support” OR “nutritional screening” OR “malnutrition” OR “sarcopenia” OR “nutritional prehabilitation” OR “oral nutritional supplements” OR “immunonutrition” OR “carbohydrate loading” OR “early oral feeding” OR “early enteral nutrition”) AND (“gastrointestinal surgery” OR “colorectal surgery” OR “gastrectomy” OR “hepato-pancreato-biliary surgery”) AND (“elective” OR “enhanced recovery” OR “ERAS”). The search covered publications from 2010 to early 2026 and was restricted to adult human studies published in English. Eligible sources included randomized controlled trials, observational studies, systematic reviews/meta-analyses, and guideline documents reporting clinically relevant perioperative outcomes (e.g., postoperative complications, infectious complications, length of stay, gastrointestinal recovery, readmissions). Exclusion criteria were pediatric populations, emergency surgery, non-gastrointestinal procedures, animal studies, and publications lacking perioperative outcome data. Titles/abstracts were screened and full texts assessed for relevance by two authors, and data were extracted using a structured approach capturing study design, surgical setting and population, nutritional intervention (type, timing, duration), comparators, and key outcomes. Given the narrative scope and heterogeneity of interventions and endpoints, findings were synthesized qualitatively, prioritizing higher-level evidence and highlighting areas of consistency as well as uncertainties across surgical contexts.

To strengthen interpretability, evidence is summarized by hierarchy (clinical practice guidelines and systematic reviews/meta-analyses, followed by randomized controlled trials and observational studies). When reporting effects, we indicate the direction of benefit and clinically relevant outcomes while acknowledging heterogeneity in surgical populations, intervention protocols (composition, timing, duration), and endpoint definitions across studies. Therefore, statements are phrased according to the certainty of evidence (e.g., “suggests”, “is associated with”, “may reduce”), and conclusions specify the contexts in which benefits are most consistently reported. Findings are interpreted through an ERAS framework, emphasizing pathway compliance, discharge criteria, and procedure-specific implementation differences.

**Figure 1:**
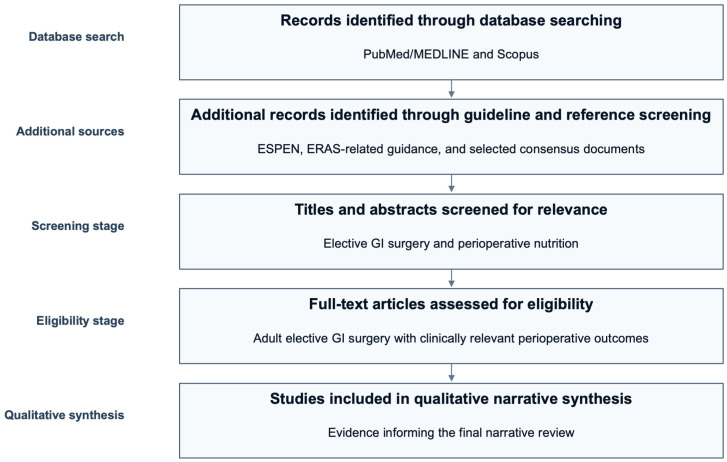
PRISMA-style study selection flow for this narrative review. This flowchart summarizes the pragmatic study selection process used for the present narrative review, including database searching, additional source identification, title and abstract screening, full-text assessment, and final inclusion in the qualitative synthesis. As this review was not designed as a formal systematic review, the figure is intended to enhance transparency regarding the literature selection process rather than to represent a protocol-driven PRISMA workflow.

Although this manuscript was designed as a narrative review rather than a formal systematic review, we sought to improve transparency regarding the study selection process. Therefore, the main stages of database searching, additional source identification, relevance screening, full-text assessment, and final qualitative inclusion are summarized in a PRISMA-style flowchart. To improve transparency regarding study identification and selection, a PRISMA-style study selection flow for this narrative review is presented in Figure 1.

## 3. Preoperative Nutritional Assessment and Risk Stratification

Nutritional assessment in preoperative elective gastrointestinal (GI) surgery is crucial, as malnutrition and muscle depletion are common and directly affect postoperative outcomes [6]. Malnutrition during preoperative phases of surgical patients is a known predisposing factor to perioperative morbidity and mortality, postoperative morbidity, infection and longer hospitalization [7]. Cancer-related cachexia, decreased oral intake, early satiety, bowel obstruction, or malabsorption is common in patients undergoing colorectal, gastric, pancreatic, or esophageal resections [8]. The prevalence of preoperative malnutrition is 17% to 20% [8]. It predisposes to infectious complications following gastrointestinal surgery, primarily in malignant diseases of the esophagus, stomach, colorectal, pancreatic and liver cancer, including inflammatory bowel diseases. It is also related to increased postoperative mortality and healthcare expenditure [7]. Therefore, systematic nutritional assessment should form part of standard preoperative evaluation rather than being reserved for visibly underweight patients.

Malnutrition, in all its forms, includes undernutrition (wasting, stunting, underweight), inadequate vitamins or minerals, overweight, obesity, and resulting diet-related noncommunicable diseases. Laboratory predictors of malnutrition include urinary creatinine and urinary 3-methylhistidine, which reflect skeletal muscle mass and fat-free mass and reflect muscle protein breakdown, but require 24 h urine collection and have poor sensitivity [9]. Other markers include serum cholesterol, delayed hypersensitivity, blood lymphocyte count, serum IGF-1, leptin, nesfatin-1 and serum zinc, which are linked to protein-energy malnutrition, immune functioning, energy homeostasis, and micronutrient deficiency, yet are commonly affected by inflammation, renal dysfunction, liver disease, and acute-phase response [9]. Contemporary diagnostic approaches rely on structured concepts such as those proposed by the Global Leadership Initiative on Malnutrition (GLIM), but in elective gastrointestinal surgery these can be operationalized pragmatically by combining phenotypic findings (Table 1) with etiologic drivers of risk (Table 2) to guide early optimization and perioperative planning.

Table 2 summarizes practical etiologic domains that commonly drive perioperative nutritional risk in elective gastrointestinal surgery, including reduced intake, impaired assimilation, and disease- or inflammation-related catabolism. In clinical workflows, these drivers should be assessed alongside phenotypic findings (Table 1) to identify patients who require targeted preoperative optimization and a predefined postoperative nutrition delivery plan.

Inflammatory burden is particularly relevant in pancreatic inflammatory disease, where severity stratification reflects the systemic catabolic impact that can compromise perioperative reserve [10].

A single-center prospective observational study conducted in 2020 and 2023 evaluated malnutrition risk among 467 elective surgery patients attending a pre-anesthetic clinic [11]. The results revealed that 19.9% of participants met the Global Leadership Initiative on Malnutrition (GLIM) malnutrition risk criteria, and 7.9% required structured preoperative nutrition [11]. It is important to note that a significant percentage of patients at risk had a body mass index (BMI) greater than 25 kg/m^2^, indicating that excess body weight does not preclude the presence of underlying nutritional deficiency. These results support the use of systematic screening instead of relying on BMI.

Sarcopenia represents a closely related but distinct risk factor characterized by loss of skeletal muscle mass and strength [12]. It can be found in patients with either normal BMI or high BMI, which is referred to as sarcopenic obesity. Sarcopenia is closely linked with postoperative morbidity, intensive care unitization, chemotherapy toxicity, and poor survival in GI oncology populations. Objective quantification of skeletal muscle mass can be achieved with computed tomography at the third lumbar vertebra (L3), which is commonly used to stage cancer [13]. The timely diagnosis of sarcopenia enables the detection of at-risk patients with necessary targeted nutritional and functional optimization in the preoperative period.

Effective perioperative care requires structured nutritional risk stratification. Routine screening should occur at the time of surgical referral to ensure early identification of at-risk patients. Besides the Global Leadership Initiative on Malnutrition (GLIM) criteria, there are other well-established nutritional risk screening tools commonly used in clinical settings to identify patients who are already malnourished or at risk. The Nutritional Risk Screening 2002 (NRS-2002) [14] is widely used in hospitals and assesses disease severity and the inability to maintain nutritional intake. Mini Nutritional Assessment Short Form (MNA 2-sf) and Long Form (MNA-LF) [15] are commonly utilized in the elderly in order to identify the early signs of nutritional decline in older adults. The Patient-Generated Subjective Global Assessment (PG-SGA) [16] is especially useful in oncology populations, which measures the history of weight, symptoms, intake, and functional capacity. The Malnutrition Screening Tool (MST) and the Malnutrition Universal Screening Tool (MUST) are straightforward, convenient tools that are based on weight loss and BMI and can thus be used in routine clinical practice [17]. In older populations, SNAQ 65+ focuses on appetite and weight loss, whereas SARC-F directly assesses sarcopenia using functional measures (strength and mobility) [18]. In gastrointestinal oncological surgery, sarcopenia has been consistently associated with higher postoperative complication rates, increased mortality, and prolonged length of stay, supporting its inclusion in perioperative risk stratification [19].

Overall, the strongest support for routine perioperative nutritional screening comes from clinical practice guidelines and consensus recommendations, complemented by observational data linking malnutrition and sarcopenia to higher postoperative morbidity and prolonged recovery. However, estimates vary across studies due to differences in diagnostic tools (e.g., GLIM components, screening instruments) and heterogeneous surgical case-mix. The clinical utility is most consistent in high-risk elective gastrointestinal surgery patients (older age, weight loss, frailty, oncologic burden), where early identification can trigger targeted nutritional optimization and perioperative planning.

## 4. Nutritional Prehabilitation: Priming the Patient

### 4.1. Timing and High-Protein Nutritional Optimization

Prehabilitation targets metabolic risk conditioning in ERAS, implying a trimodal intervention with elements of nutrition, physical exercise, and psychological stress reduction [20]. A significant decrease in complications was observed in elderly high-risk patients with American Society of Anesthesiologists (ASA) Grade III and IV [21]. The meta-analyses revealed that prehabilitation could reduce postoperative complication rates and hospital length of stay (LOS) in patients undergoing major abdominal surgery. These findings are broadly consistent across systematic reviews and meta-analyses in major abdominal surgery populations [22,23,24]. Wobith et al. [25] reported that malnutrition is an important risk factor for adverse outcomes in patients undergoing major abdominal and gastrointestinal surgery, and that nutritional prehabilitation should be targeted. The authors discovered that interventions are divided into short (7–14 days) conditioning and multimodal prehabilitation programs. However, they noted significant heterogeneity in medical nutrition therapy, which restricts the possibility of an evidence-based dietary recommendation [25].

Nutritional and exercise prehabilitation alone have been found to reduce length of hospital stay. Still, multimodal prehabilitation is reported to provide an additional effect on functional capacity, such as 6 min walking distance, even though overall effect sizes are low to very low [26]. Recent trimodal preoperative therapy (over 4 weeks) in patients undergoing colorectal resection, including targeted nutrition therapy (protein intake 1.5 g/kg/day, whey protein supplementation 30 g, and multivitamins) and exercise. The prehabilitation group showed a lower complication rate (17.1% vs. 29.7%), with an odds ratio (OR) of 0.47 (95% CI 0.26–0.87), which is a consistent benefit of multimodal prehabilitation [27].

ESPEN recommends oral nutritional supplements as protein- and energy-dense formulations to aid patients whose regular oral nutrition fails to fulfill nutritional needs, and they are regulated by the EU as Food for Special Purposes under Regulation 609/2013 [26]. ONSs contain high amounts of energy and protein, can offer a full nutritional profile, and are usually administered in 150–300 mL portions per unit, two to five times a day via a sip-feeding method to minimize gastrointestinal upset [25]. Whereas certain formulations are nutritionally balanced and can be used as a sole source of nutrition provided adequate volumes are consumed to meet individual energy and protein requirements, others can be supplemented with vitamins, trace elements or specialized fatty acids, and should be chosen based on patient needs, tolerance, and preferences [25].

Evidence from guidelines and multiple interventional studies suggests that perioperative oral nutritional supplementation—particularly when initiated preoperatively and continued postoperatively—may improve nutritional intake and is often associated with fewer complications and shorter length of stay. Reported effects are not uniform, reflecting variability in baseline nutritional risk, supplement composition and dosing, adherence, and the type and magnitude of surgery. Benefits appear most consistent in malnourished or at-risk patients undergoing major elective gastrointestinal procedures, especially within structured perioperative pathways.

### 4.2. Correcting Nutritional Deficiencies

Preoperative vitamin D deficiency may be present in up to 76%, iron deficiency in 6% to 50.5%, folic acid deficiency in 0% to 56%, low MCV in 19% to 47.9%, and anemia in 15.8% to 19.6% [28].

This malnourished condition not only impacts the quality of life of patients, but may also increase the risk of postoperative complications, such as anemia, neurological disorders, and metabolic bone disease Vitamin D has a widely acknowledged role in regulating the metabolism of calcium and phosphate, both essential to bone remodeling [29,30].

To address these deficiencies before surgery, specific interventions are recommended based on the type and severity of the deficiency. For patients with vitamin D deficiency, oral vitamin D supplements are typically prescribed, with the daily dose adjusted according to serum 25(OH)D levels [31]. The goal is to maintain serum 25(OH)D levels above 30 ng/mL [31]. For iron deficiency, oral iron supplements are usually the first-line treatment, although intravenous iron may be considered in cases of severe deficiency or poor gastrointestinal absorption [32]. Regular monitoring of serum ferritin and hemoglobin levels is essential to assess the effectiveness of the supplementation [32]. In cases of severe anemia, additional interventions such as intravenous iron or even transfusions may be necessary [33,34]. Table 3 summarizes the most commonly reported micronutrient deficiencies in metabolic and bariatric surgery, their reported preoperative prevalence among patients, and the corresponding supplementation approaches described in guideline-based or clinically oriented sources.

At the same time, micronutrient levels in blood, such as vitamin B1, vitamin B12, vitamin A, vitamin D, zinc, and copper, as well as mineral contents such as calcium, phosphorus, iron, potassium, sodium, and chloride, are measured to identify potential nutritional deficiencies [33,34].

Common preoperative dietary patterns include energy-restricted diets, low-carbohydrate ketogenic diets (LCKD), and dietary regimens incorporating ready-to-eat low-carbohydrate ketogenic products (RLCKPs) [37,38].

In addition, energy-restricted diets may increase the likelihood of eating disorders, food consumption anxiety, and internalization of weight stigma, adversely affecting pre- and postoperative outcomes [37]. Therefore, preoperative micronutrient supplementation appears particularly necessary [35,36].

When developing preoperative micronutrient supplementation strategies, patients first need to undergo a comprehensive nutritional assessment, including a detailed history, physical examination, and relevant laboratory tests, such as blood routine, serum ferritin, vitamin D, folic acid, and vitamin B12 measurements, to accurately understand the specific nutritional deficiency of patients. On this basis, a personalized supplementation program is developed according to the type and degree of micronutrients deficient in the patient. For patients with vitamin D deficiency, oral vitamin D supplements can be used, and the daily dose of supplementation depends on serum 25 (OH) vitamin D levels, and it is generally recommended to maintain serum 25 (OH) vitamin D levels above 30 ng/mL [31]. For patients with iron deficiency, oral iron or intravenous iron supplementation can be given, and changes in serum ferritin, hemoglobin and other indicators should be monitored to assess the effect of supplementation; patients with folic acid and vitamin B12 deficiency can be corrected by oral or injection of the corresponding supplement [32].

### 4.3. Immunonutrition and Integration with ERAS

Immunonutrition is an advanced ONS supplemented with arginine, omega-3 fatty acids, and ribonucleotides that targets the perioperative immune response and mitigates the effects of surgical stress on immune suppression, especially in patients with a history of major gastrointestinal (GI) cancer surgery [25]. Umbrella reviews show that exclusive preoperative administration within 5–7 days, with low heterogeneity, results in a significant reduction in postoperative infectious complications (OR about 0.52–0.58), as well as a modest decrease in hospital length of stay, but found no significant reduction in non-infectious complications or mortality [39]. They are most effective in upper GI, colorectal, and esophageal resections, independent of baseline nutritional status, and have also been validated within ERAS protocols, for example, in the SONVI trial, where infectious complication reduction was significant despite a constant overall hospital stay [40]. When immunonutrition was compared with a control group using hypercaloric, hypernitrogenous supplements during the seven days of preoperative intervention and up to five days of postoperative intervention, the length of stay did not differ. Nevertheless, patients undergoing immunonutrition showed a reduction in the number of complications, with the greatest reduction in infectious complications (23.8 vs. 10.7, *p* = 0.0007) [40].

Recent evidence syntheses provide further support for perioperative immunonutrition in major gastrointestinal surgery, while also reinforcing the need for cautious interpretation. An umbrella review and a network meta-analysis both reported that immunonutrition may reduce postoperative morbidity, particularly infectious complications, in visceral surgery, although the magnitude of benefit varies according to formulation, timing, route, and surgical setting [41,42]. These findings are consistent with the broader view that immunonutrition is best interpreted as a protocol- and population-dependent intervention rather than a universally effective perioperative measure.

Procedure-specific data also help refine the clinical applicability of immunonutrition. In upper gastrointestinal cancer surgery, a recent meta-analysis found a significant reduction in infectious complications with perioperative immunonutrition, supporting its use in selected higher-risk cohorts [43]. In contrast, within ERAS-based colorectal practice, the SONVI multicenter randomized trial highlighted that the incremental benefit of immunonutrition over standard oral supplementation should be interpreted within the broader context of pathway-based perioperative care, where baseline nutritional support and ERAS compliance may already attenuate between-group differences [44].

The evidence base for immunonutrition includes meta-analyses and randomized trials showing potential reductions in infectious complications and, in some settings, shorter length of stay, although effect sizes differ across surgical domains and protocols. Heterogeneity is driven by differences in formulations, timing (preoperative-only vs. perioperative), duration, and patient selection. The most reproducible signals of benefit are reported in major gastrointestinal oncologic surgery and in patients with higher baseline risk, whereas routine use in low-risk elective cases remains less certain.

Overall, the most defensible interpretation is that immunonutrition may offer the greatest value in major GI oncologic surgery and other higher-risk settings, whereas routine use in lower-risk elective pathways remains less certain and should be individualized.

## 5. Preoperative Carbohydrate Loading and Fasting Concepts

The traditional ‘nil-by-mouth after midnight’ approach has been progressively replaced by evidence-informed fasting policies within ERAS pathways. Table 4 summarizes the main links between surgical stress, insulin resistance, and perioperative hyperglycemia, together with practical modifiable perioperative levers. Pro-inflammatory cytokines (IL-6, TNF-α) and sympathoadrenal (catecholamines, cortisol) mediators are released in response to surgical stress and suppress the GLUT4 transporter in skeletal muscle and activate hepatic gluconeogenesis. In parallel, fasting increases glucagon release and systemic insulin resistance [45]. The resultant effect is a hyperglycemic environment that is severe and affects wound healing and recovery. Possible mechanisms to counteract this insulin resistance include modern perioperative interventions, such as preoperative carbohydrate loading, which improve the stability of metabolic processes and postoperative outcomes. Such interventions consequently decrease morbidity, hospital stays and expenses. In elective abdominal surgery cohorts managed within ERAS pathways, controlled studies and meta-analyses suggest that preoperative carbohydrate loading may improve early recovery metrics and patient comfort, although effect sizes vary across protocols and endpoints.

### 5.1. Metabolic Rationale

Loading with carbohydrate (CHO), as is most often 400 mL of a 12.5% maltodextrin solution two hours before surgery, is an effective way to shift patients to the fed metabolic state, which otherwise would be hyperglycemic and insulin-resistant following surgery [46]. This metabolic transition lowers the catabolic impulse that burns glycogen stores and triggers muscle protein degradation, thereby creating functional reserve.

### 5.2. Functional Advantage

CHO loading has been shown to reduce thirst, hunger, and anxiety, preoperative factors that may increase and contribute to preoperative discomfort and affect intra-operative hemodynamics, as well as biochemical parameters [47]. Further, a series of randomized controlled trials has shown that preoperative CHO consumption is associated with a significant difference in the interval to bowel motility restoration, an outcome of special importance in abdominal surgery, where postoperative ileus increases length of stay and morbidity [48,49].

### 5.3. Safety Profile

The concern that clear fluids may elevate the risk of pulmonary aspiration is not well grounded in clinically stable patients undergoing elective, non-obstructed procedures, rather than emergency or obstructed contexts [50]. The systematic reviews and meta-analyses support the idea that clear, non-alcoholic liquids administered to patients up to two hours before induction do not increase the rate of aspiration in contrast to more rigorous fasting protocols [51]. This safety profile is explained by the rapid gastric emptying of clear fluids, the low administered volume, and the absence of solids that would delay emptying or increase residual volume.

The metabolic and functional data are strong, but due to a variety of study designs, differences in CHO concentration, volume, timing, and patient demographics, extrapolation should be done with caution. In addition, the possibility of individual differences in gastric emptying, especially in diabetic or motility-disordered subjects, demands individualized fasting plans. Another issue is that preoperative clear fluid intake increases logistical challenges for operating room workflow and patient education, which need to be considered in the development of institutional policy [52]. The evidence supports a paradigm shift toward a more differentiated form of midnight fasting, including the use of CHO loading [53]. This is an approach that ensures congruence between metabolic physiology and clinical outcomes, minimizes patient suffering, and safeguards the safety of elective and non-obstructed operations. The next generation of research in this area should standardize CHO loading protocols, expand indications to include high-risk surgical patients, and incorporate these practices into improved recovery pathways to optimize perioperative treatment.

Guidelines and controlled studies generally support preoperative carbohydrate loading as part of ERAS, with evidence suggesting improved patient comfort and reduced perioperative insulin resistance, and in some studies earlier gastrointestinal recovery. Nevertheless, impacts on “hard” outcomes (overall complications, length of stay) are less consistent, partly due to differences in CHO regimens, fasting policies, and concurrent ERAS elements. Benefits are most applicable to non-diabetic or well-controlled diabetic patients undergoing elective procedures, while careful selection is required in patients with delayed gastric emptying or high aspiration risk.

To summarize the main perioperative nutritional strategies discussed so far—together with the typical hierarchy of evidence, the most reproducible clinical signals, and the populations most likely to benefit—Table 5 provides an at-a-glance synthesis intended for clinical interpretation. Importantly, these effects remain context- and protocol-dependent across procedures and baseline risk profiles (Table 5).

**Figure 2:**
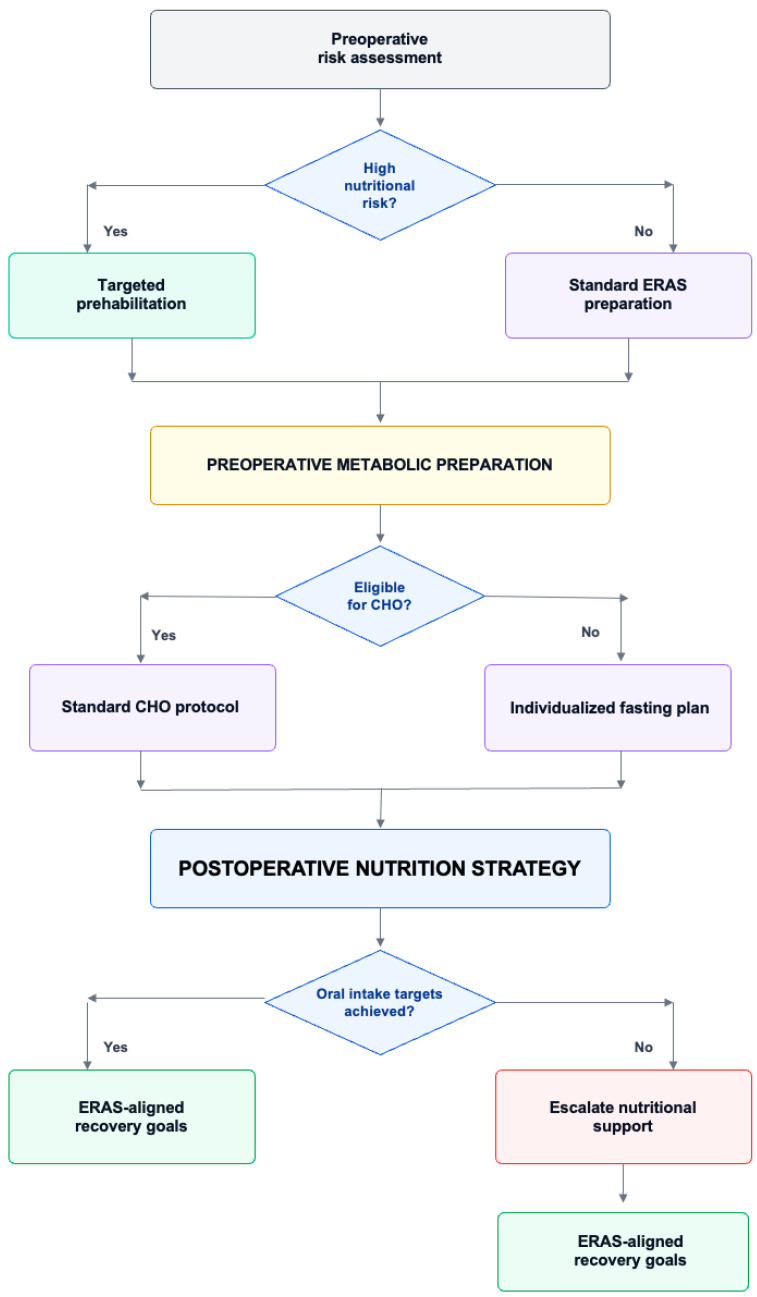
ERAS-focused perioperative nutrition pathway for elective gastrointestinal surgery. This schematic summarizes a pragmatic, stepwise perioperative nutrition pathway within ERAS-focused care, linking early nutritional risk assessment to prehabilitation, preoperative metabolic preparation, and postoperative oral feeding advancement. It emphasizes major decision nodes related to nutritional risk, carbohydrate-loading eligibility, achievement of oral intake targets, and escalation when nutritional goals are not met. The figure is intended as a practical conceptual framework and should be interpreted according to procedure-specific context and baseline patient risk. Abbreviations: CHO, carbohydrate loading; ERAS, Enhanced Recovery After Surgery.

For practical implementation, Figure 2 summarizes an ERAS-focused, stepwise workflow linking early risk stratification to perioperative nutrition delivery decisions and escalation triggers.

## 6. Early Postoperative Feeding: Rationale and Protocols

### 6.1. Rationale for Early Feeding

The shift away from the traditional ‘rest-and-gut’ doctrine toward early feeding is supported by evidence that enteral nutrient exposure and specific dietary components can preserve mucosal integrity and gut barrier function, thereby limiting translocation of luminal microbial products.

Current guideline-based evidence continues to support early postoperative oral feeding as the preferred nutritional approach after major surgery whenever clinically feasible. The 2025 ESPEN update reinforces the principles of avoiding unnecessary postoperative fasting, re-establishing oral intake as early as possible, and initiating nutritional therapy promptly when nutritional risk is evident, while also emphasizing that implementation must remain individualized according to surgical procedure, tolerance, and risk profile [54]. Accordingly, early feeding should be interpreted as a structured ERAS-aligned strategy rather than a uniform one-size-fits-all intervention.

Table 6 outlines proposed mechanisms through which early postoperative feeding may influence gastrointestinal recovery and downstream clinical correlates, highlighting heterogeneity in the certainty of evidence across contexts. These elements also influence mucosal immunity through microbiome modulation, suppressed LPS-TLR4/ERK signaling, decreased ROS, and excessive immune cell activation. SCFAs from fermentable fibers also enhance additional barrier function and anti-inflammatory mechanisms [55]. Together, these nutrients play a concerted role in maintaining epithelial integrity and immune response balance, thus demonstrating their therapeutic benefit in gastrointestinal diseases. Together, these mechanisms may support epithelial integrity and a more balanced mucosal immune response, although clinical impact remains context-dependent across procedures and feeding protocols.

Building on these gut barrier and microbiome-mediated mechanisms, perioperative microbiome-directed strategies—particularly lactic acid bacteria (LAB)-based probiotics and synbiotics—have been investigated as adjuncts to nutritional care to reduce dysbiosis, support mucosal integrity, and potentially mitigate postoperative infectious morbidity.

Traditional prolonged postoperative fasting has been linked to mucosal atrophy, poor motility, and an increased risk of postoperative complications such as infection and ileus. Biomarkers of gut permeability, such as plasma lipopolysaccharide-binding protein, can be used to show that perioperative feeding within the initial 24 h of abdominal surgery suppresses endotoxin leakage [56]. Together, these mechanisms may support epithelial integrity and a more balanced mucosal immune response, although clinical impact remains context-dependent across procedures and feeding protocols.

### 6.2. Microbiome-Directed Adjuncts: Probiotics/Synbiotics (Lactic Acid Bacteria)

Improving surgical outcomes requires optimal nutritional management. Increasing attention is being given to the gut microbiome as a modifiable factor in postoperative outcomes, alongside macronutrient adequacy and immunonutrition. Surgical stress, anesthesia, antibiotic prophylaxis, bowel preparation and perioperative fasting disrupt intestinal microbial balance, contributing to increased gut permeability, systemic inflammation and infectious complications [57].

Lactic acid bacteria (LAB), including strains within the genera *Lactobacillus*, *Pediococcus* and *Bifidobacterium*, represent a nutritional approach that is targeted to reduce perioperative dysbiosis. Their integration into surgical nutrition care may support intestinal barrier function, modulate immune responses and reduce postoperative morbidity [58,59].

#### 6.2.1. Mechanisms of Action of Lactic Acid Bacteria in Surgical Nutrition

The potential benefits of lactic acid bacteria in surgical nutrition care are supported by multiple interconnected core mechanisms linked to molecular, immunological and metabolic pathways. These mechanisms are particularly relevant in the context of surgical stress, barrier disruption or antibiotic exposure and are directly targeting the pathophysiological drivers of postoperative infectious complications [60,61].

#### 6.2.2. Preservation of Intestinal Barrier Function

Surgical stress and systemic inflammation can disrupt epithelial tight junctions increasing intestinal permeability and facilitating bacterial translocation. This process contributes to systemic inflammatory response and surgical site infections. LAB support barrier integrity by upregulating tight junction proteins, enhancing mucin production or by reducing lipopolysaccharide translocation. By increasing epithelial cohesiveness and preserving the mucus layer, LAB may prevent microbial translocation during the perioperative period. This barrier-protective effect is one of the most clinically relevant mechanisms underlying reduced infectious complications in abdominal surgery [62,63].

#### 6.2.3. Modulation of Immune Responses

Surgery induces complex immune responses characterized by concomitant hyperinflammation and transient immunosuppression. An increased vulnerability to infection and organ dysfunction is the result of dysregulated cytokine signaling. LAB interact with intestinal epithelial cells and immune cells via pattern recognition receptors influencing downstream signaling pathways. This interaction can: reduce pro-inflammatory cytokines (TNF-α, IL-6, IL-1β), increase anti-inflammatory mediators (IL-10), enhance secretory IgA production in the gut. Through balanced immunomodulation rather than broad immune stimulation, LAB may attenuate excessive inflammatory responses while preserving antimicrobial defense [64,65].

#### 6.2.4. Limitation of Pathogen Overgrowth

After the administration of antibiotics during surgery, opportunistic pathogens, such as *Enterobacteriaceae* species, may be able to occupy ecological niches in the gut. Studies have shown that pathogen overgrowth is strongly associated with postoperative infectious morbidity. To combat this, LAB produce antimicrobial metabolites, such as lactic acid, bacteriocins—strain-specific peptides with targeted antimicrobial activity and hydrogen peroxide, which have the capacity to inhibit growth of pathogenic organisms, including Gram-negative bacteria implicated in surgical site infections. Other mechanisms include adhesion to epithelial surfaces—LAB compete with pathogens for epithelial binding sites, reducing colonization potential and quorum sensing interference—emerging data suggest that some LAB strains may disrupt pathogen quorum sensing systems, thereby attenuating virulence factor expression [66,67].

#### 6.2.5. Short-Chain Fatty Acid (SCFA) and Metabolic Signaling

Although LAB are primarily lactate producers, they contribute also to SCFA production. When fermented by gut microbes, fibers produce short-chain fatty acids (SCFAs) like butyrate, propionate, and acetate, which have anti-inflammatory effects and help maintain gut barrier integrity [68,69]. Butyrate serves as a primary energy substrate for colonocytes and enhances epithelial regeneration. It also promotes tight junction assembly and mucosal repair. Moreover, SCFAs act as inhibitors for histone deacetylases (HDACs), altering gene transcription toward anti-inflammatory pathways. In the postoperative state—characterized by insulin resistance and catabolism—these metabolic interactions may contribute to improved glycemic stability and mucosal healing.

#### 6.2.6. Gut–Organ Axes in Surgical Recovery

The gut microbiome interacts with distant organs via systemic metabolites and immune signaling (e.g., gut–brain axis—affecting stress responses and recovery trajectories) LAB-mediated modulation of these axes may partially explain reductions in infectious and inflammatory complications observed in clinical trials [68].

#### 6.2.7. Clinical Evidence in Surgical Populations

The clinical evidence for perioperative LAB-based probiotics and synbiotics derives primarily from randomized controlled trials and meta-analyses, with outcomes influenced by substantial heterogeneity in strain composition, dosing, timing (preoperative-only vs. perioperative), duration, antibiotic exposure, baseline nutritional risk, and the extent to which supplementation is embedded within bundled ERAS care. Quantitative syntheses nevertheless support a clinically meaningful signal in selected settings. A meta-analysis of randomized controlled trials in elective abdominal surgery reported a significant reduction in postoperative infectious complications with perioperative probiotics or synbiotics (RR approximately 0.56), while colorectal surgery-specific meta-analyses found reductions in overall infectious complications and surgical site infections, with relative risk estimates generally ranging from 0.55 to 0.70 depending on the endpoint analyzed [70,71,72,73,74,75]. These pooled findings, together with additional procedure-specific reports, indicate that the strongest and most reproducible clinical signal currently lies in elective colorectal surgery.

Outside colorectal surgery, the evidence base remains narrower but still suggestive. An umbrella review of 11 meta-analyses in colorectal cancer surgery further confirmed a favorable signal for lower overall infection rates and surgical site infections [74], whereas a recent meta-analysis in major liver surgery reported reductions in postoperative infections and antibiotic exposure, suggesting that benefit may extend to selected higher-risk hepatobiliary settings, although the supporting data remain less extensive than in colorectal cohorts [76]. By contrast, evidence in esophageal and gastric surgery remains comparatively limited and emerging; available studies suggest potential improvements in postoperative nutritional tolerance, selected pulmonary outcomes, and inflammatory response profiles in specific cohorts, but generalizability is constrained by smaller sample sizes and protocol variability [77]. Overall, these data support a risk- and procedure-stratified interpretation: probiotics and synbiotics appear most promising in major abdominal surgery with higher infectious risk and/or greater anticipated dysbiosis burden, whereas their incremental value in lower-risk elective pathways remains less certain and likely strain-specific.

#### 6.2.8. Safety Considerations

In elective surgery cohorts, LAB-based probiotics and synbiotics are generally well tolerated, but safety remains contingent on appropriate patient selection, product quality, and clinical context. The practical goal is not routine use in all patients, but cautious consideration in settings where potential benefit is biologically plausible and patient risk is acceptable.

Heightened caution is warranted in severely immunocompromised patients (e.g., post-transplant recipients and those receiving intensive chemotherapy), in sepsis or multi-organ failure (where use—if considered at all—should occur only under close monitoring), and in patients with significant intestinal compromise (e.g., severe mucosal injury or conditions that may increase microbial translocation risk) [78]. Although probiotic-associated bacteremia has been reported, the overall incidence appears low when clinically validated preparations are used appropriately. When integrated as an adjunct to—rather than a substitute for—structured nutritional optimization and ERAS principles, LAB supplementation may contribute to reduced infectious morbidity and support recovery in selected major abdominal surgery populations, while acknowledging that observed benefits are not universal and remain dependent on strain and protocol characteristics [73,76]. Rigorous patient selection, adherence to clinically validated strains, and monitoring in high-risk contexts are key safeguards [78].

The most reproducible clinical signals are currently seen in elective colorectal surgery, whereas evidence in other GI and hepatobiliary procedures remains more limited and should be interpreted with greater caution.

Remaining uncertainties include optimal strain selection, dosing, timing, and the patient subgroups most likely to benefit, which should be clarified by future procedure- and risk-stratified trials.

For practical interpretation, Table 7 summarizes the proposed roles of perioperative LAB-based probiotics/synbiotics, indicates how consistently these signals have been reported in the literature, identifies settings where benefit is most plausible, and delineates key safety boundaries for patient selection (Table 7).

### 6.3. Clinical Evidence for Early Postoperative Oral Intake

Early oral intake within 24 h has become standard practice in many colorectal procedures. Recent evidence syntheses further clarify that the clinical effects of early postoperative feeding are procedure-dependent. In elective colorectal surgery, a 2025 systematic review and meta-analysis reported fewer total complications, shorter time to first flatus, and reduced length of stay with early oral feeding, although vomiting occurred more frequently and careful advancement remained important [79]. In bowel surgery more broadly, a recent systematic review highlighted that while most patients tolerate early oral feeding, a meaningful proportion may not do so until postoperative day 4, and the clearest benefit appears to be shorter hospitalization among those who tolerate intake early [80]. In upper gastrointestinal malignancy, a 2024 network meta-analysis suggested that early oral feeding is safe and effective overall but also indicated that the optimal timing may differ by endpoint, with postoperative day 3 ranking favorably for safety and effectiveness [81].

The randomized controlled trials and systematic reviews all indicate that early feeding decreases the occurrence rates of postoperative ileus, shortens the LOS in hospitals, and enhances patient satisfaction without escalating adverse events. As an illustration, colorectal resection multicenter RCT reported a 30% decrease in ileus and a 1.5-day decrease in LOS in patients who commenced oral intake within 24 h as compared to those who followed a later schedule [82]. Similarly, meta-analyses involving the diverse types of gastrointestinal surgeries have also demonstrated that early oral intake has been linked to a reduced incidence of surgical site infections and reduced readmissions further emphasizing its safety profile. Notably, such benefits are preserved among subgroups of patients, such as those with comorbid conditions, such as diabetes or obesity, as long as perioperative glucose control is satisfactory.

### 6.4. Real-Life Procedures and Dietetic Interventions

Universally, EN is more desirable than parenteral nutrition because it is associated with reduced infections, cost-effectiveness, and functionality of the gut. PN is used on patients with a non-functional gastrointestinal tract or patients who are unable to obtain at least 60 per cent of their caloric requirements through EN or oral intake seven days after surgery [83]. The feeding protocols in the practical stages usually go through clear liquids, all liquids, soft solids, and to regular diet as tolerated. Early EN is often administered using jejunostomy or nasojejunal feeding tubes in upper gastrointestinal surgeries including esophagectomy to circumvent the proximal anastomosis to decrease anastomotic stress. Jejunal feeding can help ensure postoperative nutritional delivery and is often associated with earlier return of bowel function in upper gastrointestinal procedures. Reported effects on anastomotic leak are variable across studies and depend on procedure type, feeding protocols, and baseline anastomotic risk; therefore, jejunal feeding is best framed as a supportive strategy within a broader perioperative pathway rather than a stand-alone determinant of leak prevention [84]. Tolerance is assessed regularly as protocols include abdominal distension, nausea and residual volume monitoring, and individualized escalation is allowed. If feeding intolerance has developed, step-down measures such as re-introduction of clear liquids or trophic feeds are observed and escalation is reinitiated when the intolerance has ended.

Across guidelines and interventional evidence, early oral intake and/or EN is generally feasible and may accelerate gastrointestinal recovery, with many studies reporting no increase in major complications compared with traditional delayed feeding. However, outcomes vary by procedure type (upper GI vs. colorectal vs. HPB), anastomotic risk profile, and definitions of “early feeding,” and intolerance rates are not negligible in selected cohorts. The clearest applicability is within ERAS pathways for elective colorectal and other lower-risk GI resections, whereas individualized advancement is prudent after complex upper GI or high-risk anastomoses.

Overall, the most defensible interpretation is that early postoperative feeding should be considered standard within ERAS-oriented care, but its advancement should remain procedure-specific and tolerance-guided, particularly after upper GI and other higher-risk resections.

## 7. Clinical Outcomes and Functional Recovery

### 7.1. Postoperative Complications

Nutritional optimization is a key component of perioperative care, particularly in older adults undergoing major elective abdominal surgery. Evidence from systematic review and meta-analytic data suggests that preoperative oral nutritional supplementation may reduce postoperative infectious complications, with the most consistent signal reported for surgical site infections (SSIs) [85]. The background process is the restoration of the micronutrients and amino acids which promote the growth of immune cells, generation of cytokines and integrity of the barrier affecting the dampening of the systemic inflammatory response leading to infection predisposition. In addition to infection, the recent data highlight the great impact of preoperative provision of targeted nutritional support on the reduction in postoperative delirium, which is a frequent and expensive complication of aging. In this way, nutritional optimization acts in two forms of protection, namely, strengthening of host protection against pathogens and stabilization of neurocognitive homeostasis, which are the most important factors in safe surgical recovery.

### 7.2. Ileus, Anastomotic Leaks, and Healing

Prolonged postoperative ileus (PPOI) remains a clinically relevant driver of delayed recovery after major colorectal surgery, supporting the need for structured prevention and early recognition strategies [49]. Nutritional status also has an effect on intestinal motility and anastomotic integrity, but this is less direct. Sufficient protein consumption and a set of micronutrients including zinc, and vitamin C provide an environment in which collagen synthesis and angiogenesis take place and are required to repair tissues [86]. Nevertheless, the key factors that cause anastomotic leak are the surgical technique, tension of the anastomosis, and local perfusion; the secondary but non-negligible role is played by nutrition. The increased leakage associated with prolonged malnutrition is due to the inhibition of fibroblast proliferation and undermining of the mucosal barrier functions, weakening the anastomosis [87]. On the other hand, in case of corrected malnutrition, the number of leaks is not reduced similarly but the level of leaks and the morbidity decreases. In addition, the best nutrition accelerates the recovery of postoperative ileus through maintenance of enteric neural activity and motilin secretion, which shortens the bowel rest period and related complications. Thus, although nutrition is not a panacea of surgical leakage, a proper nutritional condition is an essential supplement that aids healing and minimizes the risk of adverse sequelae development.

### 7.3. Length of Stay, Readmissions, and Functional Recovery

Structured nutritional programs embedded within ERAS pathways are consistently associated with shorter postoperative length of stay across elective gastrointestinal procedures, although the magnitude of benefit varies by surgical population, institutional baseline practice, and protocol adherence.

Recent evidence syntheses further support the view that ERAS-related outcome gains depend not only on pathway adoption, but also on implementation quality. A large meta-analysis of randomized clinical trials reported that ERAS guidelines were associated with shorter length of stay and fewer complications overall, whereas effects on hospital readmission were not significant, and outcome estimates varied according to the number of ERAS elements delivered and the type of surgery performed [88]. These findings support the interpretation that compliance and implementation fidelity are central determinants of the observed clinical effect rather than peripheral methodological details.

Across elective gastrointestinal procedures, baseline comorbidity burden and frailty influence complication risk and discharge readiness, reinforcing the rationale for risk-stratified ERAS pathway implementation and patient-centered discharge criteria [89].

Given the variability in protocol compliance, discharge criteria, case-mix, and endpoint definitions across studies and centers, Table 8 summarizes key sources of heterogeneity that influence observed effects on length of stay and readmissions and indicates how consistently these factors have been described in the literature (Table 8).

Implementation heterogeneity is also increasingly recognized as a major explanation for between-study variability. An umbrella review focusing on gastrointestinal procedures found that ERAS programs were associated with reduced surgical site infection but not with a significant effect on 30-day readmission, while also emphasizing substantial variation in protocol compliance and standardization across studies [90]. More recent studies have similarly shown that higher compliance is associated with faster recovery, earlier discharge, and improved short-term outcomes in upper gastrointestinal surgery, reinforcing the importance of adherence when interpreting pathway effectiveness [91]. In addition, emerging international data suggest that ERAS compliance is associated with shorter length of stay and fewer complications across hospitals and procedure types, further supporting its role as a major implementation-sensitive outcome driver [92].

Taken together, these findings indicate that ERAS-related benefits should be interpreted not only in relation to pathway design, but also according to how consistently and completely pathway elements are implemented in routine practice.

Beyond length of stay and readmission metrics, patient-centered outcomes such as quality of recovery, functional capacity, and health-related quality of life are increasingly recognized as important endpoints in ERAS-oriented perioperative care. These dimensions may capture clinically meaningful benefits that are not always fully reflected by traditional surgical outcomes alone. Recent ERAS-based data in minimally invasive gastric surgery, for example, suggest that structured pathway implementation may improve the quality of early postoperative recovery, supporting the inclusion of patient-reported and functional recovery measures in perioperative nutrition research [93].

Functional recovery can also be assessed using objective and patient-centered tools, including the 6 min walk test, early recovery scores, and validated quality-of-life instruments. Recent evidence suggests that multimodal prehabilitation may improve functional capacity, particularly as reflected in 6 min walk test performance, although these gains are not always paralleled by uniform improvements across all postoperative endpoints [94,95]. In addition, ERAS compliance itself appears to influence recovery-oriented outcomes, including earlier return of oral intake and gastrointestinal function after esophageal and gastric surgery, reinforcing the importance of implementation quality when interpreting patient-centered results [91].

Overall, ERAS pathways are supported by substantial guideline endorsement and a broad body of comparative studies, which consistently show an association with shorter postoperative length of stay in elective gastrointestinal surgery. Nevertheless, the magnitude of benefit varies across institutions and procedures, reflecting differences in baseline care, adherence to ERAS elements, discharge criteria, and patient complexity. Readmission effects are more heterogeneous across studies, and improvements are most consistently observed when high protocol compliance is achieved, and perioperative nutrition is integrated within a multidisciplinary pathway [88]. These reductions may be explained by several synergistic effects, including lower infection rates, less postoperative delirium, earlier return of bowel function, and improved wound healing.

Notably, patient-centered outcomes have become a central component of modern ERAS initiatives because functional capacity and quality of life (QoL) are increasingly regarded as being as important as conventional morbidity indicators. The six-minute walk test is a validated measure of aerobic endurance and lower-limb function and is commonly used in perioperative research as an objective marker of postoperative functional recovery. Some studies report that preoperative nutritional counseling and supplementation are associated with modestly better postoperative functional performance, including an approximately 15–20 m greater distance on the six-minute walk test compared with control groups; however, effect sizes vary with baseline nutritional risk, surgical magnitude, and concurrent prehabilitation components [96]. Significant improvements in physical functioning, role limitation, and overall well-being are also found in nutritionally optimized cohorts in QoL assessments, which are usually quantified through disease-specific instruments, i.e., the EORTC QLQ-C30, or generic ones, such as the SF-36 [97]. These patient-reported outcomes provide evidence of the holistic effect of nutrition: in addition to complication prevention, patients may recover independence more effectively and maintain better psychosocial well-being. Overall, the most informative interpretation is that perioperative nutritional strategies within ERAS pathways should be evaluated not only by complications and hospital stay, but also by their effect on recovery quality, functional capacity, and other patient-centered outcomes.

## 8. Conclusions: Practical Takeaways and Future Directions

In elective gastrointestinal surgery, perioperative nutritional optimization has become a central component of ERAS-aligned care pathways. Across the available literature, routine screening for nutritional risk and reduced functional reserve supports early identification of high-risk patients and facilitates targeted optimization strategies, including oral nutritional supplementation, selected immunonutrition protocols, carbohydrate loading when appropriate, and early postoperative feeding within structured pathways. Importantly, the magnitude and consistency of clinical benefit varies by procedure type and baseline patient risk, and effects are most reproducible when nutritional strategies are embedded within high-compliance, multidisciplinary perioperative programs.

This review has limitations. The evidence base is heterogeneous with respect to intervention definitions (timing, composition, dosing, and duration), surgical case-mix (upper GI, colorectal, HPB), and outcome reporting, and many studies evaluate nutrition as part of bundled ERAS care, making it difficult to isolate the independent effect of single components. Additional limitations include variable baseline nutritional risk across cohorts, inconsistent adherence reporting, and differences in discharge criteria that influence length of stay outcomes. As a narrative review based on a focused rather than fully systematic search strategy, this manuscript is inherently subject to some degree of selection bias and uneven representation of the available literature, despite efforts to improve transparency through structured screening and a PRISMA-style study selection flow. Future systematic reviews, scoping reviews, and procedure-specific meta-analyses will be important to further strengthen the evidence base in this field.

Future research should prioritize procedure-specific, risk-stratified trials using standardized nutrition targets and transparent adherence reporting. Key PICO-oriented research questions include: (1) in malnourished or sarcopenic patients scheduled for major elective GI resection (P), does a structured multimodal prehabilitation program combining high-protein supplementation and resistance training (I), compared with standard ERAS care alone (C), reduce infectious complications and improve functional recovery at 30 days (O)?; (2) in patients undergoing elective colorectal resection (P), does protocolized early oral intake with defined escalation triggers (I), compared with traditional diet advancement (C), reduce prolonged postoperative ileus and shorten length of stay without increasing readmissions (O)?; and (3) in high-risk GI oncology patients receiving neoadjuvant therapy (P), does perioperative immunonutrition administered for a standardized perioperative window (I), compared with isocaloric/isoproteic standard supplementation (C), reduce infectious complications and support timely adjuvant therapy delivery (O)?

## Figures and Tables

**Table 1:** Phenotypic domains relevant to perioperative malnutrition risk in elective gastrointestinal surgery (GLIM-informed): practical assessment and action.

Phenotypic Domain (What You Observe)	How to Assess in Routine Practice (Examples)	Red Flags/Interpretation (Pragmatic)	Why It Matters Perioperatively (Clinical Relevance)	Suggested Perioperative Response (Actionable)
**Unintentional weight loss/adverse weight trajectory**	Serial weights (EHR trend), patient report (time course), clothing fit, corroboration from caregivers	Clear downward trend, rapid loss over weeks–months, or persistent loss despite intake	Signals depleted reserves and catabolic state; associated with higher infectious complications, impaired wound healing, and prolonged recovery	Early dietitian referral; start high-protein ONS; address drivers (pain, nausea, obstruction, dysphagia); consider prehabilitation if time allows
**Low body reserves/reduced energy stores (contextual body size)**	BMI as context (not a stand-alone diagnosis), body habitus history, clinical exam (loss of subcutaneous fat), frailty features	“Low reserve” phenotype (thin/frail), or sarcopenic obesity suspected (normal/high BMI but poor strength/function)	Low reserve → poor tolerance to surgical stress; sarcopenic obesity can mask risk and is linked to worse outcomes	Individualized protein/energy targets; avoid prolonged fasting; integrate nutrition plan into ERAS; monitor intake vs. targets
**Reduced muscle quantity or quality (sarcopenia phenotype)**	Opportunistic CT-derived muscle estimates when available; BIA/ultrasound (where available); mid-upper arm circumference (proxy); clinical exam	Low muscle proxy, visible wasting, poor endurance; discordance between BMI and strength/function	Predicts complications, longer length of stay, delayed mobilization and functional recovery	Protein-forward strategy; resistance exercise component (prehab); early mobilization; plan early postoperative nutrition delivery
**Reduced muscle strength and/or functional performance**	Handgrip dynamometry, chair-stand test, SARC-F, gait speed, 6-minute walk test (if feasible)	Low strength/function relative to age/sex norms; inability to complete basic functional tests; fatigue limiting ADLs	Captures functional reserve; correlates with postoperative disability, prolonged recovery, and discharge challenges	Multimodal prehabilitation (nutrition + exercise + optimization); set discharge goals; consider post-acute support planning
**Frailty-linked low physiologic resilience (phenotypic expression)**	Brief frailty screen (e.g., Clinical Frailty Scale), history of falls, exhaustion, low activity; interpreted alongside domains above	Frailty features plus any phenotype above amplifies risk	Frailty interacts with malnutrition/sarcopenia → higher morbidity, slower recovery, higher readmission risk	Multidisciplinary planning (surgery–anesthesia–nutrition–physio); prioritize preoperative optimization; consider geriatric input when available

This table provides an original, practical perioperative framework summarizing phenotypic domains commonly used to identify patients at risk of malnutrition and poor functional reserve in elective gastrointestinal surgery. It is GLIM-informed but presented as an operational tool rather than a reproduction of consensus criteria or cut-off values.

**Table 2:** Etiologic domains relevant to perioperative malnutrition risk in elective gastrointestinal surgery (GLIM-informed): clinical triggers and practical responses.

Etiologic Domain (Driver)	How It Presents Clinically (Examples)	Common Elective GI Surgery Contexts	What It Implies for Nutrition Delivery	Suggested Perioperative Response (Actionable)
**Reduced oral intake (quantity/quality)**	Early satiety, anorexia, pain, nausea/vomiting, dysphagia, poor dentition, fatigue; low appetite scores	Upper GI malignancy, benign strictures, severe GERD/achalasia, frail elderly	Likely failure to meet protein/energy targets orally without structured support	Early dietitian plan; high-protein ONS; symptom control; meal fortification; consider short prehab window if time allows
**Impaired assimilation/malabsorption**	Chronic diarrhea/steatorrhea, bloating; nutrient deficiencies; weight loss despite intake	Chronic pancreatitis, small bowel disease, IBD, cholestasis, post-gastrectomy/bypass physiology	Oral intake may be ‘adequate’ yet ineffective; higher risk of micronutrient deficits	Treat underlying cause; targeted micronutrients; enzyme replacement when indicated; lower threshold for EN if targets unmet
**Inflammatory burden/disease stress–related catabolism**	Elevated CRP, active malignancy, systemic inflammation; cachexia phenotype	GI oncology, advanced inflammatory disease, chronic infection/inflammation states; pancreatic inflammatory disease (including severe pancreatitis phenotypes) [10]	Higher protein needs and accelerated lean-mass loss; risk persists even with acceptable BMI	Protein-forward targets; minimize unnecessary fasting; early postoperative feeding where feasible; integrate within ERAS
**Treatment-related nutrition compromise**	Chemo-related anorexia/dysgeusia, mucositis, steroids, fatigue; reduced activity	Neoadjuvant pathways for GI cancer, prolonged preoperative treatment windows	Intake and function decline during ‘waiting period’ → missed optimization opportunity	Proactive prehabilitation (nutrition + exercise); timed supplementation; multidisciplinary coordination
**Procedure-related constraints and anticipated postoperative intake limitation**	Expected delayed gastric emptying, intolerance risk, planned anastomosis protection strategies; prolonged NPO risk	Esophagectomy, gastrectomy, major HPB resections	Higher likelihood of postoperative underfeeding unless route is planned	Predefine postoperative pathway (oral vs. EN); consider feeding access when appropriate; set intake milestones and escalation triggers
**Socio-functional barriers influencing intake and adherence**	Low health literacy, poor support at home, financial barriers, depression, substance misuse	Any elective GI population (especially frail/older adults)	Plans fail without adherence/support despite appropriate prescribing	Simplify regimen; involve caregivers; written plan; early follow-up; connect with social support services

Legend: This table is an original perioperative synthesis of common etiologic drivers of malnutrition risk in elective gastrointestinal surgery. It is GLIM-informed at the conceptual level but intentionally avoids reproducing consensus grading tables or cut-off thresholds.

**Table 3:** Common micronutrient deficiencies and recommended supplementation strategies in metabolic and bariatric surgery.

Micronutrient	Reported Preoperative Prevalence Among Patients	Recommended Supplementation
**Vitamin D**	Up to 76% of patients [28]	Oral vitamin D supplements to maintain serum 25(OH)D levels above 30 ng/mL [31]
**Iron**	6% to 50.5% of patients [28]	Oral or intravenous iron, depending on severity and absorption [32]
**Folic Acid**	0% to 56% of patients [28]	Oral folic acid supplements [32]
**Vitamin B12**	Frequently reported as clinically significant in relevant studies [33,34]	Oral or injectable vitamin B12 [32]
**Zinc**	Frequently reported as clinically significant in relevant studies [33,34]	Oral zinc supplements [35,36]
**Vitamin A**	Frequently reported as clinically significant in relevant studies [33,34]	Oral vitamin A supplements [32,35,36]
**Vitamin E**	Frequently reported as clinically significant in relevant studies [33,34]	Oral vitamin E supplements [32,35,36]

Note: Percentages refer to the reported prevalence of each deficiency among patients in the cited studies, not to the proportion of reviewed articles. Reported ranges reflect heterogeneity across study populations, procedures, baseline nutritional risk, and diagnostic thresholds.

**Table 4:** Surgical stress, insulin resistance, and perioperative hyperglycemia: pathway and modifiable levers.

Pathway Component	Key Mediators/Contributors (Examples)	Physiological Effect	Clinical Correlate in Elective GI Surgery	Modifiable Perioperative Levers (Practical)
**Neuroendocrine stress response to surgery**	Catecholamines, cortisol, glucagon; sympathetic activation	Hepatic glucose output ↑; peripheral glucose uptake ↓	Early postoperative hyperglycemia; glucose variability	Minimize unnecessary fasting; consider preoperative carbohydrate drink when appropriate; avoid excessive dextrose infusions; standardized ERAS workflow
**Inflammation-driven insulin resistance**	Pro-inflammatory cytokines; acute-phase response	Insulin signaling impairment; skeletal muscle glucose uptake ↓	Hyperglycemia despite “normal” preop status	Early mobilization; multimodal analgesia to reduce stress burden; prompt nutrition delivery when tolerated
**Catabolic metabolism and lean mass loss**	Proteolysis, lipolysis; reduced physical activity	Muscle protein breakdown ↑; functional reserve ↓	Delayed functional recovery; prolonged LOS	Protein-forward nutrition strategy; early oral/enteral intake; structured physiotherapy; consider prehabilitation in high-risk patients
**Iatrogenic contributors**	High-dose steroids (when used), parenteral glucose load, perioperative hypothermia, overly liberal fluids	Hyperglycemia risk ↑; insulin requirement ↑	Higher glucose excursions; increased monitoring needs	Review glucose-containing fluids; protocolized insulin therapy per local policy; maintain normothermia; rational fluid strategy
**Downstream clinical associations**	Immune dysfunction (neutrophil function), impaired collagen synthesis, endothelial dysfunction	Susceptibility to infection and impaired wound repair (associative evidence)	Higher-risk profile for SSI and other infectious complications; delayed healing	Perioperative glycemic targets per institutional protocol; integrate nutrition + glycemic monitoring into ERAS pathway; prioritize high-risk groups (diabetes, frailty, malnutrition)

Legend: This table is an original synthesis summarizing commonly described mechanisms linking surgical stress to perioperative hyperglycemia and highlighting practical, modifiable perioperative levers. It is intended for conceptual and clinical workflow guidance rather than as a quantitative model; **Abbreviations:** ↑, increase/increased; ↓, decrease/decreased.

**Table 5:** Perioperative nutritional interventions in elective GI surgery: evidence hierarchy, expected benefits, and best-fit populations.

Intervention	Evidence Hierarchy (Typical)	Most Reproducible Clinical Signal	Consistency of Reporting in the Literature	Where Benefit Is Most Likely	Key Limitations/Heterogeneity	Practical Implementation Note
**Nutritional screening (malnutrition/sarcopenia)**	Guidelines + observational cohorts	Risk stratification; identifies high-risk	Consistently reported	Elderly, oncologic, weight loss, frailty	Tool variability; sarcopenic obesity	Screen early (first surgical consult); trigger dietitian plan
**ONS (high-protein)**	Guidelines + mixed RCTs/meta-analyses	Lower infectious morbidity/LOS (often)	Frequently reported	Malnourished/at-risk; major resections	Dose/adherence varies; baseline risk matters	Start preop, continue postop; monitor intake vs. targets
**Immunonutrition**	Meta-analyses + RCTs	Infectious complications ↓ in some settings	Variably reported	GI oncology; higher-risk major surgery	Formulation/timing vary; not universal	Use protocolized window + defined formula; avoid blanket use
**CHO loading**	Guidelines + controlled studies	Comfort, insulin resistance ↓; GI recovery earlier in some	Variably reported	Non-diabetic or controlled diabetic; low aspiration risk	Hard endpoints inconsistent; protocol differences	Standardized CHO drink; follow fasting guidelines
**Early oral/EN feeding**	Guidelines + RCTs/meta-analyses	GI recovery earlier; no ↑ major complications in many cohorts	Consistently reported in colorectal surgery; variably reported in upper GI/HPB surgery	Colorectal, lower-risk GI resections	Procedure-dependent; intolerance possible	Define advancement milestones + escalation triggers

Legend: Table 5 provides an ERAS-focused, at-a-glance synthesis of perioperative nutritional strategies in elective gastrointestinal surgery. Interventions are summarized by typical evidence hierarchy (guidelines/meta-analyses, randomized trials, observational studies), the most reproducible clinical signals, the consistency with which effects have been reported in the literature, and the patient populations in whom benefit is most consistently described. Interpretation is framed within pathway implementation factors—including protocol compliance, discharge criteria, and procedure-specific context—which contribute to heterogeneity in reported outcomes. Note: “Consistency of reporting in the literature” is expressed qualitatively to reflect how reproducibly a given issue or intervention effect has been described across guidelines, evidence syntheses, randomized trials, and observational studies; **Abbreviations:** ↑, increase/increased; ↓, decrease/decreased.

**Table 6:** Early postoperative feeding: proposed mechanisms, clinical correlates, and certainty of evidence.

Mechanistic Domain	Representative Mediators/Processes (Examples)	Expected Direction of Effect	Clinical Correlate in Elective GI Surgery	Certainty/Evidence Profile (Pragmatic)	Key Limitations/Notes
**Mucosal integrity and barrier function**	Tight junction regulation; epithelial turnover; enterocyte energy supply; short-chain fatty acid signaling	Permeability ↓; barrier resilience ↑	Reduced “feed intolerance cascade” in some settings; potentially lower infectious signal (procedure-dependent)	Mixed: strong mechanistic rationale; clinical outcomes heterogeneous	Effects depend on procedure, timing, and tolerance; bundle effects within ERAS
**Motility and gastrointestinal recovery**	Enteral stimulation of gut–brain axis; neurohormonal responses; reduced ileus drivers	Earlier transit; earlier return of bowel function	Earlier flatus/stool; earlier progression of diet; earlier discharge readiness	Moderate: supported by many clinical protocols but variable results	Intolerance not negligible; upper GI/complex resections often require slower advancement
**Inflammation and immune modulation**	Reduced stress signaling with feeding; mucosal immune homeostasis; microbiome-immune interactions	Inflammatory tone ↓ (hypothesis); immune function supported	Potential reduction in infectious complications in some cohorts	Low–moderate: indirect clinical signal, often confounded	Difficult to isolate feeding effect from ERAS compliance and case-mix
**Microbiome and metabolite profile**	Maintenance of luminal substrate; microbial diversity support; SCFA production	More favorable microbial metabolites; colonization resistance ↑	Improved stool patterns and tolerance in some contexts	Low–moderate: mechanistic and associative clinical data	Highly variable between patients; antibiotics and bowel prep confound
**Protein delivery and muscle preservation**	Earlier achievement of protein targets; reduced fasting-related catabolism	Lean mass loss ↓; functional recovery ↑	Improved mobilization and rehabilitation participation	Moderate: depends on actual intake achieved	Requires monitoring of intake vs. targets; nausea/pain can limit delivery
**Anastomotic healing context**	Adequate substrate delivery; avoidance of prolonged NPO; local perfusion context	Neutral-to-beneficial overall when tolerance is respected	Early feeding is feasible after many colorectal procedures; more caution in high-risk anastomoses	Procedure-dependent	High-risk anastomoses (complex upper GI/HPB) may need individualized advancement

Legend: This table is an original synthesis outlining commonly proposed mechanisms through which early postoperative feeding may influence recovery. “Certainty/evidence profile” is a pragmatic descriptor (conceptual to moderate) reflecting heterogeneity across procedures, protocols, and endpoints; it does not represent a formal GRADE assessment; **Abbreviations:** ↑, increase/increased; ↓, decrease/decreased.

**Table 7:** Perioperative probiotics/synbiotics (LAB): potential roles, target populations, and safety boundaries.

Topic	Key Message	Consistency of Reporting in the Literature	Best-Fit Populations	Where Evidence Is Weaker	Safety Boundary (“Avoid/Caution”)
**Clinical signal**	Infectious complications may decrease in some cohorts; effects strain/protocol-dependent	Frequently reported in colorectal surgery; limited/emerging in other GI procedures	Colorectal surgery; higher infectious risk	Low-risk elective cases; non-colorectal procedures	N/A
**Mechanistic rationale**	Barrier support + immune modulation + dysbiosis mitigation (conceptual)	Consistently supported conceptually	Major abdominal surgery with dysbiosis burden	Hard outcomes not uniform	N/A
**Implementation**	Adjunct, not substitute for ONS/ERAS; needs protocol	Variably reported	Centers with defined product/strain and timing	Unstandardized “over-the-counter” use	N/A
**Safety**	Generally well tolerated in elective cohorts	Frequently discussed; limited by high-risk subgroup data	Stable elective patients	Critically ill cohorts	Avoid/caution: severe immunosuppression, sepsis/MOF, significant mucosal compromise

Legend: Table 7 summarizes the proposed roles of perioperative LAB-based probiotics/synbiotics, the surgical settings in which benefit appears most plausible, and the main safety boundaries relevant to patient selection. In addition, the table indicates how consistently each topic has been described in the literature, acknowledging the heterogeneity of strains, protocols, and surgical populations. Note: “Consistency of reporting in the literature” is expressed qualitatively to reflect how reproducibly each topic has been described across randomized trials, meta-analyses, mechanistic studies, and clinically oriented reviews; **Abbreviations:** LAB, lactic acid bacteria; ONS, oral nutritional supplements; ERAS, Enhanced Recovery After Surgery; N/A, not applicable.

**Table 8:** Why ERAS effects vary: drivers of heterogeneity in LOS and readmission outcomes.

Domain	What Varies Across Studies/Centers	How It Biases LOS/Readmissions	Consistency of Reporting in the Literature	Practical Mitigation in Interpretation
**Protocol compliance**	Adherence to key ERAS elements (feeding, mobilization, analgesia, fluids)	High compliance → clearer LOS reduction; low compliance → diluted effect	Consistently reported	Report/consider compliance; interpret bundled effects cautiously
**Discharge criteria**	Formal discharge rules vs. clinician judgment	Shorter LOS may increase early readmission if discharge is premature	Frequently reported	Interpret LOS alongside readmission and functional readiness
**Case-mix**	Procedure type (upper GI vs. colorectal vs. HPB), oncologic burden, frailty	Higher complexity → smaller/variable LOS effects	Consistently reported	Stratify conclusions by procedure/risk
**Outcome definitions**	Different definitions for ileus, complications, “tolerance”	Apparent inconsistency across trials	Frequently reported	Prefer standardized endpoints; state definition used
**Baseline care**	“Control care” may already be ERAS-like	Smaller incremental gains	Variably reported	Compare context; avoid deterministic language

Legend: Table 8 summarizes common sources of heterogeneity that influence the observed effects of ERAS pathways on length of stay and readmission outcomes in elective gastrointestinal surgery. In addition to describing what varies across studies and how such variation may bias outcome interpretation, the table indicates how consistently each source of heterogeneity has been reported in the literature. Note: “Consistency of reporting in the literature” is expressed qualitatively to reflect how reproducibly each source of heterogeneity has been described across comparative ERAS studies, evidence syntheses, and guideline-informed discussions.

## Data Availability

No new data were created or analyzed in this study.

## Abbreviations

The following abbreviations are used in this manuscript:

ADLs	Activities of Daily Living
BIA	Bioelectrical Impedance Analysis
CHO	Carbohydrate (carbohydrate loading)
EHR	Electronic Health Record
GLUT4	Glucose Transporter Type 4
LAB	Lactic Acid Bacteria
MNA	Mini Nutritional Assessment
MST	Malnutrition Screening Tool
MUST	Malnutrition Universal Screening Tool
NPO	Nil Per Os (nothing by mouth)
NRS-2002	Nutritional Risk Screening 2002
ONS	Oral Nutritional Supplements
PG-SGA	Patient-Generated Subjective Global Assessment
PICO	Population, Intervention, Comparator, Outcome
QLQ-C30	EORTC Quality of Life Questionnaire—Core 30
SARC-F	Strength–Assistance with walking–Rise from a chair–Climb stairs–Falls questionnaire
SCFA	Short-Chain Fatty Acid
SF-36	36-Item Short Form Health Survey
SNAQ 65+	Short Nutritional Assessment Questionnaire 65+
